# The Autism Phenome Project: Toward Identifying Clinically Meaningful Subgroups of Autism

**DOI:** 10.3389/fnins.2021.786220

**Published:** 2022-01-17

**Authors:** Christine Wu Nordahl, Derek Sayre Andrews, Patrick Dwyer, Einat Waizbard-Bartov, Bibiana Restrepo, Joshua K. Lee, Brianna Heath, Clifford Saron, Susan M. Rivera, Marjorie Solomon, Paul Ashwood, David G. Amaral

**Affiliations:** ^1^MIND Institute, University of California, Davis, Davis, CA, United States; ^2^Department of Psychiatry and Behavioral Sciences, School of Medicine, University of California, Davis, Davis, CA, United States; ^3^Center for Mind and Brain, University of California, Davis, Davis, CA, United States; ^4^Department of Psychology, University of California, Davis, Davis, CA, United States; ^5^Department of Pediatrics, School of Medicine, University of California, Davis, Davis, CA, United States; ^6^Department of Medical Microbiology and Immunology, School of Medicine, University of California, Davis, Davis, CA, United States

**Keywords:** autism, MRI, heterogeneity, immune, development, gastrointestinal, ERP, females

## Abstract

One of the most universally accepted facts about autism is that it is heterogenous. Individuals diagnosed with autism spectrum disorder have a wide range of behavioral presentations and a variety of co-occurring medical and mental health conditions. The identification of more homogenous subgroups is likely to lead to a better understanding of etiologies as well as more targeted interventions and treatments. In 2006, we initiated the UC Davis MIND Institute Autism Phenome Project (APP) with the overarching goal of identifying clinically meaningful subtypes of autism. This ongoing longitudinal multidisciplinary study now includes over 400 children and involves comprehensive medical, behavioral, and neuroimaging assessments from early childhood through adolescence (2–19 years of age). We have employed several strategies to identify sub-populations within autistic individuals: subgrouping by neural, biological, behavioral or clinical characteristics as well as by developmental trajectories. In this Mini Review, we summarize findings to date from the APP cohort and describe progress made toward identifying meaningful subgroups of autism.

## Introduction

Autistic individuals present with a broad continuum of social communication difficulties as well as non-social characteristics such as repetitive behaviors, intense focused interests and sensory experiences. Co-occurring medical, developmental, and mental health conditions are common (American Psychiatric Association, 2013; Soke et al., 2018). This heterogeneous presentation has led to inconsistency in research findings and challenges with identifying etiological causes and optimal treatments or interventions. One approach to constraining heterogeneity is to restrict research samples (e.g., all male or IQ cut offs). However, this has led to underrepresentation of certain portions of the autism spectrum, including autistic females (Lai et al., 2015) and individuals with intellectual disability (Russell et al., 2019), thus limiting generalizability of findings.

Another approach to addressing heterogeneity is to conduct comprehensive evaluations of all autistic individuals and then stratify based on one or more salient characteristics. These subgroups can then be evaluated further for shared etiology and mechanistic underpinnings. The ultimate goal is to decrease variability in treatment response among autistic individuals by identifying individualized interventions specific to the phenotypic and biological commonalities of given subgroups. Here, we summarize findings from the Autism Phenome Project (APP), a large, longitudinal, multidisciplinary study that has utilized this approach to identify autism subgroups at the behavioral, neural, and biological levels.

The APP was initiated in 2006 to integrate behavioral, neuroimaging, and other biological and medical data in a large cohort of autistic children with the overarching goal of identifying clinically meaningful subgroups that share common biological or behavioral features. Children with autism are enrolled at 2–3.5 years. Age and sex-matched non-autistic children with no developmental delay are enrolled as typically developing (TD) controls. Thus far, APP participants have been followed for 4 time points through early and middle childhood, with the oldest participants currently returning for a fifth time point during adolescence. Initial recruitment was conducted from 2006 to 2011 with over 300 children enrolled during that timeframe, which clarifies that enrollment did not stop at 300 participants: enrollment of new participants is ongoing.

Diagnostic confirmation for autism is conducted by licensed psychologists using the Autism Diagnostic Observation Schedule (ADOS) (Lord et al., 2000, 2012) and Autism Diagnostic Interview -Revised (Lord et al., 1994). TD control participants are screened for autism using the Social Communication Questionnaire (Rutter et al., 2003) and developmental delay using the Mullen Scales of Early Learning (MSEL) (Mullen, 1995). Initial recruitment reflected the male predominance of autism diagnoses, and about 4–5 times more boys than girls were enrolled.

To increase female representation within the APP cohort, we initiated the Girls with Autism—Imaging of Neurodevelopment (GAIN) study in 2014. The study protocol is identical to the APP, and all participants in the GAIN study are automatically included in the APP dataset. Thus far, GAIN participants have completed three early childhood time points and are scheduled to return for a 4th middle childhood timepoint. With almost 100 autistic females in the cohort, evaluation of similarities and differences across sexes are now possible and included in all analyses.

**FIGURE 1:**
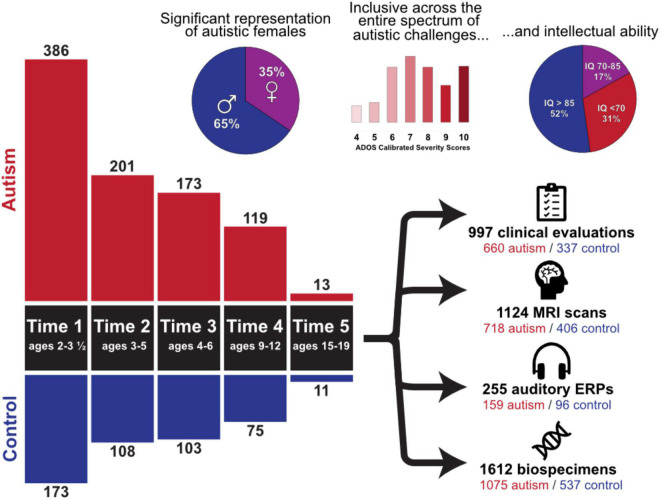
Overview of the Autism Phenome Project cohort. Longitudinal behavioral, neuroimaging, and medical data has been acquired since 2006 in over 500 children from early childhood through adolescence. The cohort includes children across the entire autism spectrum, including understudied groups such as females and children with co-occurring intellectual disability. Data collection at all-time points is ongoing. ADOS Calibrated Severity Scores at Time 1 and IQ scores at Time 4 are depicted.

**FIGURE 2:**
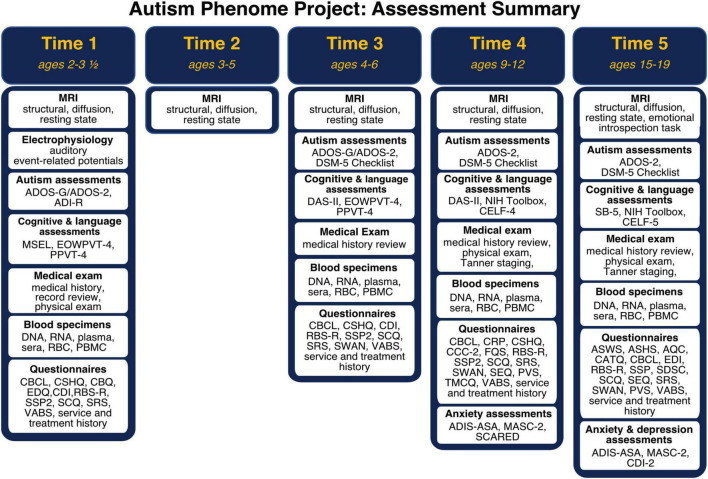
Autism Phenome Project longitudinal study design and assessment battery at each time point from early childhood through adolescence. ADI-R, Autism Diagnostic Interview; ADIS-ASA, Anxiety Disorders Interview Schedule with Autism Addendum; ADOS, Autism Diagnostic Observation Schedule; AQC, Alexithymia Questionnaire for Children; ASWS, Adolescent Sleep Wake Scale; ASHS, Adolescent Sleep Hygiene Scale; CATQ, Camouflaging Autistic Traits Questionnaire; CBCL, Child Behavior Checklist; CBQ, Children’s Behavior Questionnaire; CCC-2, Children’s Communication Checklist; CDI-2, Child Depression Inventory; CDI, MacArthur-Bates Communicative Development Inventories; CELF, Clinical Evaluation of Language Fundamentals; CRP, Child Rearing Practices; CSHQ, Children Sleep Habits; DAS-II, Differential Ability Scale—2nd Edition; EDI, Emotional Dysregulation Inventory; EDQ, Early Development Questionnaire; EOWPVT, Expressive One Word Picture Vocabulary Test; FQS, Friendship Quality Scale; MASC-2, Multidimensional Anxiety Scale for Children; MSEL, Mullen Scales of Early Learning; PBMC, peripheral blood mononuclear cells; PPVT, Peabody Picture Vocabulary Test; PVS, Self-Perception Profile for Children and Peer Victimization Scales; RBC, red blood cells; RBS-R, Repetitive Behavior Scale—Revised; SB-5, Stanford Binet—Fifth Edition; SCARED, Screen for Child Anxiety Related Disorders; SCQ, Social Communication Questionnaire; SDSC, Sleep Disturbances Scale for Children; SEQ, Sensory Experiences Questionnaire; SRS, Social Responsiveness Scale; SSP2, Short Sensory Profile 2; SWAN, Strengths and Weaknesses of Attention-Deficit/Hyperactivity-symptoms and Normal-behaviors; TMCQ, Temperament in Middle Childhood; VABS, Vineland Adaptive Behavior Scale.

Participant demographics across all timepoints are summarized in Figure 1. Importantly, the cohort includes children with all levels of intellectual functioning, including 30% of participants with IQs in the range of intellectual disability at the middle childhood time point (9–12 years of age). Figure 2 depicts the longitudinal assessment battery, which includes comprehensive behavioral assessments, medical exams, and magnetic resonance imaging (MRI). Auditory event related potentials (ERPs) were conducted at Time 1. Medical exams are conducted by developmental pediatricians and include assessment of pubertal status at later time points and characterization of gastrointestinal symptoms and family histories of autoimmune conditions. Blood specimens from the child and biological parents are used to evaluate immune function and genomic sequencing. MRIs are conducted during natural nocturnal sleep for the first three time points (Nordahl et al., 2008), resulting in inclusion of children with all levels of developmental ability. To ensure that all could be followed longitudinally, we developed strategies to acquire MRI scans in children with intellectual disability while awake at the older time points (Nordahl et al., 2016).

Below, we highlight efforts from 30 published studies utilizing this comprehensive, multidisciplinary dataset to identify subgroups based on neural, other biological, behavioral/clinical characteristics, and developmental trajectories. In many instances, subgroups ascertained on the basis of one characteristic (e.g., neural) are cross validated using characteristics from other disciplines (e.g., behavioral) to identify multidisciplinary commonalities and increase clinical significance.

## Subgrouping by Neural Characteristics

### Brain Volume

Because of multiple reports of early brain enlargement (Courchesne et al., 2001; Sparks et al., 2002; Hazlett et al., 2005), we were initially surprised by the degree of overlap in brain volume between autistic children and controls. It was clear early on that not all autistic children have enlarged brains and that group mean differences were driven by a small subset of autistic children with cerebral volumes outside the range of their age-matched TD peers. We began evaluating clinical characteristics of this subset of children with larger brain volumes (i.e., megalencephaly) and found that 22% of males who had a regressive onset of autism had megalencephaly compared to only 5% of males without regression (Nordahl et al., 2011).

Subsequent efforts to define this subgroup accounted for height in order to distinguish brain enlargement from generalized somatic overgrowth (Klein et al., 2013; Campbell et al., 2014). Autism with disproportionate megalencephaly (ASD-DM) was defined as a ratio of cerebral volume to height greater than 1.5 standard deviations above age- and sex-matched TD controls. In the APP, 12.6% of autistic boys and 6% of autistic girls were characterized as ASD-DM at Time 1. Clinical characteristics of this subgroup include lower language ability at age 3 and higher rates of intellectual disability at age 6 (Amaral et al., 2017). Further, ASD-DM is not simply a uniformly bigger brain, but rather has distinct regional expansion of surface area (Ohta et al., 2016) and gyrification patterns (Libero et al., 2019) that differ from autistic children without DM. The rate of cerebral gray and white matter growth in ASD-DM does not differ from other autistic children, and cerebral volume remains elevated in this subgroup throughout early and middle childhood (Libero et al., 2016; Lee et al., 2021). The ASD-DM subgroup also has differentially altered auditory ERP responses, exhibiting a different pattern of loudness-dependent electrophysiological responses than other autistic children (De Meo-Monteil et al., 2019).

### Extra-Axial Cerebrospinal Fluid

The role of cerebrospinal fluid (CSF) in neurodevelopment and disease is an area of increased focus (Lehtinen et al., 2013; Shen, 2018). Elevated extra-axial CSF, sometimes referred to as external or communicating hydrocephalus, is characterized by excessive CSF in the subarachnoid space between the brain and dura mater. Although commonly considered benign in children under 2 years, recent evidence from two independent infant sibling cohorts suggests that elevated extra-axial CSF during infancy is associated with increased likelihood for autism diagnosis at age 3 (Shen et al., 2013, 2017). In the APP cohort at Time 1, 13% of autistic children had elevated levels extra-axial CSF (Shen et al., 2018). This subset also had increased sleep problems compared to autistic children without elevated extra-axial CSF.

### Auditory Event-Related Potentials

Prior studies have examined whether, on average, autistic and TD groups differ in latencies and amplitudes of auditory ERPs (reviewed by Williams et al., 2021). However, few have examined whether different sub-populations within autism show differential auditory responses cf. (Salmond et al., 2007; De Meo-Monteil et al., 2019; Roberts et al., 2019). In the APP, autistic participants exhibit—at the group mean level—diminished amplitudes of the N2, a negative-going cortical response to auditory stimuli over frontocentral channels ∼200–350 ms post-stimulus, at Time 1 (Dwyer et al., 2021a), consistent with prior studies (Williams et al., 2021). However, examination of inter-individual differences in ERP morphologies suggests this pattern is driven by a subset of participants with atypical positive-going ERP responses over the spatiotemporal window associated with the N2 (Dwyer et al., 2021c). Although the behavioral implications of this ERP positivity are unclear, this finding illustrates how group averages can distort and occlude patterns at the individual and subgroup levels.

We have also examined how ERPs in autism are affected by differences in auditory stimulus intensity. We clustered autistic and TD participants based on the relative strengths of ERP global field power responses to 50 through 80 dB tones (Dwyer et al., 2020). Autistic participants with disproportionately strong responses to loud (80 dB) tones were reported by caregivers to struggle with auditory distractibility, which we interpret as a neurophysiologic reflection of hyperacusis or noise distress. Intriguingly, relative to TD controls, a disproportionate number of autistic participants with higher cognitive ability scores than other autistic participants formed part of another cluster characterized by gradual increases in response amplitude from 50 to 70 dB.

## Subgrouping by Other Biological Characteristics

### Biological Sex

Biological sex is an important source of variability in autism. As a first step, we have evaluated autistic females as a biologically defined subgroup based on sex assigned at birth. We recognize, however, that autistic females do not represent a singular, distinct phenotype of autism. Additional studies are needed to explore heterogeneity within autistic females and gender diverse populations.

Thus far, we have identified sex differences in diffusion-weighted properties and in the organization of the corpus callosum (Nordahl et al., 2015; Andrews et al., 2019). At Time 1, autistic females had a smaller callosal region projecting to anterior frontal regions compared to TD females. In contrast, autistic males had a smaller callosal region projecting to orbitofrontal cortex than TD males (Nordahl et al., 2015). More globally, autistic females exhibited a slower rate of cerebral gray and white matter growth across early childhood relative to sex-matched controls, but no difference in overall cerebral volume at any time point (Lee et al., 2021). In contrast, autistic males did not differ in rate of cerebral growth from TD male controls, but they had larger cerebral brain volumes across early childhood than TD males, an effect driven by the subgroup with disproportionate megalencephaly, which is much less common in autistic females.

Behaviorally, autistic males and females in the APP do not differ on measures of core autistic traits or developmental ability at Time 1. Autistic females were, however, more highly represented in a subgroup with clinically significant levels of co-occurring mental health symptoms (40% of females compared to 22% of autistic males) (Nordahl et al., 2020). Moreover, amygdala volume was associated with internalizing and externalizing problems in autistic girls, but not boys, suggesting sex differences in the role of the amygdala in autism. Sex differences in amygdala functional connectivity are also apparent during early childhood (Lee et al., 2020).

### Immune Factors

Evidence supporting dysregulation of the immune system in autism includes a higher prevalence of familial autoimmunity, gestational immune influences, as well as altered innate and adaptive immune responses in some autistic individuals (reviewed in Hughes et al., 2018). We have investigated several immune-related factors in the APP, including evaluation of specific immune cell types and response to immunological stressors. In one study, peripheral blood mononuclear cells from Time 1 plasma samples were stimulated with the bacteria product lipopolysaccharide (LPS). A subset of 44% of autistic children had an increased pro-inflammatory profile that was associated with lower developmental scores and increased sleep problems and aggression (Careaga et al., 2017b). Altered cell signaling in autistic children was also related to immune activation and repetitive behaviors (Onore et al., 2017). In a related study of the innate immune system, which includes cell types that respond to LPS, the frequency of myeloid dendritic cells was increased by 25% in autistic children and associated with gastrointestinal problems, repetitive behaviors, and amygdala volumes (Breece et al., 2013).

We also investigated levels of cell adhesion molecules that immune cells use to tether to endothelial cells before gaining access to tissue, including brain parenchyma. There were decreased levels of platelet endothelial adhesion molecule-1 (PECAM-1) in plasma from autistic children. In support of the notion that altered trafficking of immune cells may have implications in brain homeostasis, PECAM-1 levels were positively correlated with head circumference in TD controls, but not in autistic children (Onore et al., 2012).

Maternal immune factors have also been linked to neurodevelopmental disabilities (Careaga et al., 2017a). In the APP, 8% of autistic children were born to mothers with a specific set of maternal IgG autoantibodies that bind to fetal brain tissue, compared to none of the TD controls. This subset also exhibited 12% larger brain volume than controls and 7% larger brain volume than other autistic children (Nordahl et al., 2013). More recently we found that maternal immune conditions such as autoimmunity, asthma and allergies that occurred during pregnancy were predictors of externalizing behaviors in autistic children (Patel et al., 2020). Maternal asthma was the most commonly reported condition and was twice as common in mothers of autistic males (20%) than autistic females (11%).

### Gastrointestinal Symptoms

Gastrointestinal (GI) concerns are frequently reported by parents of autistic children and may be related to immune dysregulation (Buie et al., 2010). This is particularly concerning because it may be more challenging for autistic children to verbalize or communicate physical pain, leading to lack of appropriate medical care. Within the APP, we evaluated parent-reported GI symptoms at Time 1 and identified a subgroup comprising 48% of autistic children who experience significant GI problems (Restrepo et al., 2020). Children with significant GI problems also had higher levels of self-injurious behaviors, restricted stereotyped behaviors, sensory sensitivities, aggressive behavior, attention problems, as well as sleep problems such as shorter sleep duration, night awakenings, and parasomnia.

## Subgrouping by Behavioral and Clinical Characteristics

### Anxiety

In the APP, children are assessed for clinical anxiety disorders at middle childhood (Time 4) and adolescence (Time 5) using the Anxiety Disorders Interview Schedule-IV-Parent Interview (ADIS) (Albano and Silverman, 1996) to identify traditional DSM forms of anxiety including generalized anxiety disorder (GAD), separation anxiety, specific phobia, and social phobia. The ADIS Autism Spectrum Addendum (ADIS-ASA) (Kerns et al., 2017) is administered to identify anxieties distinctly related to autism, including idiosyncratic fears, fear relating to social confusion, intense interest fears, and fears of change. At Time 4, 69% of autistic children were diagnosed with clinically significant anxiety, with 21% having a DSM anxiety disorder, 17% an ADIS-ASA distinct anxiety disorder, and 31% both (Kerns et al., 2020). Differences in the rates of DSM-anxiety presentations in autistic children with and without intellectual ability were also noted. Autistic children with intellectual disability predominately endorsed specific phobias while other DSM anxieties were less common compared to autistic children without intellectual disability.

### Language

While some autistic individuals learn and use language consistent with their chronological age, others experience delayed or impaired language development. At Time 1, we grouped autistic children based on language ability and examined associations with white matter development (Naigles et al., 2017). Autistic children were grouped into a low (48%; language beginning or not yet begun), middle (21%; language included a stable lexicon of nouns), or high (31%; language included a large vocabulary plus some grammar) groups. Subgroup differences were identified in the left and right inferior longitudinal fasciculus (ILF), left superior longitudinal fasciculus, and the left corticospinal tract. In particular, fractional anisotropy in the occipital region of the ILF was correlated with language ability, but not ADOS severity scores. Other efforts utilizing the APP have identified subgroups based interactions between social communication and language development (Blume et al., 2021) and grammatical language ability (Wittke et al., 2017).

## Subgrouping by Developmental Trajectories

### Cognitive Development

Intellectual functioning is one of the most heterogeneous aspects of autism (Maenner, 2020). In the APP, we identified subgroups based on the trajectory of intellectual functioning across early childhood (Times 1–3) (Solomon et al., 2018). Four distinct trajectories were identified: two groups, comprising 26 and 18% of the sample, respectively, had IQs in the intellectual disability range at both time points; a third group (22%) had IQs in the normal range at both time points. Of particular interest, a fourth group, comprising 35% of the cohort, initially had IQs in the intellectual disability range but made significant gains (34 points) to have IQs in the normal range by age 6–7.

### Sensory Behaviors

Atypical sensory experiences and behaviors have been reported in 82–97% of autistic people (Dellapiazza et al., 2018) and are related to diminished quality of life (Lin and Huang, 2019; McConachie et al., 2020). In the APP, the Short Sensory Profile (SSP; McIntosh et al., 1999) was used to examine sensory behaviors at Times 1 and 3. Almost two-thirds of autistic participants showed a stable intense sensory phenotype characterized by high levels of atypical sensory behavior at both time points; these participants also had elevated anxiety levels (Dwyer et al., 2020). Another third of autistic participants and almost all TD participants exhibited milder, more typical sensory behaviors at both time points.

Another study examined how different SSP subscales (from the factor solution of Williams et al., 2018) contribute to overall SSP trajectories in autism and typical development (Dwyer et al., 2021b). Almost 28% of autistic participants showed disproportionately high levels of low energy/weakness, most likely reflecting hypotonia. Interestingly, these participants had higher cognitive ability scores at Time 1 relative to other autistic participants, which suggests that hypotonia might be developmentally protective in autism. Around 13% of autistic participants showed intensely atypical sensory behaviors across all subscales of the SSP as well as ERP hyper-responsivity to loud sounds. Autistic participants in these hypotonic and generalized-intense subgroups had more anxiety and sleep disturbances than the remaining subgroup that exhibited less intense sensory behaviors.

### Autism Characteristics

Recent evidence suggests that the intensity or degree of autistic characteristics can vary over time (Gotham et al., 2012). Using change in ADOS calibrated severity scores (CSS) from Time 1 to Time 3, we found that 54% of APP participants had stable autism characteristics while 29% significantly decreased and 17% increased in ADOS-CSS scores over this period. Change groups did not differ by initial ADOS-CSS or hours of intervention received. However, the group with decreasing ADOS-CSS had higher IQ scores and were more likely to be female (Waizbard-Bartov et al., 2021). Follow up MRI analyses between these groups identified that individuals with increasing degree of autism characteristics had slower development of the sagittal stratum fiber bundle (Andrews et al., 2021).

## Conclusion and Future Directions

The studies described above reveal subpopulations within the broad autism spectrum that are likely obscured when group-level comparisons between autistic and TD control groups are made. Efforts to identify subgroups with more homogenous characteristics provides a deeper characterization of the heterogeneity of autism and co-occurring conditions. Importantly, the identification of subgroups is not meant to divide or marginalize portions of the autism community. Rather, some promising subgrouping efforts may guide clinical care by influencing selection of interventions or access to services and supports. For example, disproportionate megalencephaly at age 3 may provide early clues to parents about children who may require higher levels of support, or individuals with autism distinct forms of co-occurring anxiety may benefit from autism-specific anxiety interventions. In other cases, subgrouping may increase awareness for, and treatment of, debilitating co-occurring medical conditions such as gastrointestinal dysfunction. These subgroups need to be further examined and validated in order to make specific recommendations for clinical care. A recent review of subtyping efforts in autism research provides a checklist for validating subtypes that will be useful for future studies (Agelink van Rentergem et al., 2021).

To fully achieve the goals of the APP, future studies will require cohorts representative of all autistic individuals, including all cognitive abilities, speaking and non-speaking individuals, all racial and ethnic groups, and increased representation of female and gender diverse individuals. The current APP sample size may be an adequate starting point for subgroup identification, but much larger sample sizes are necessary. Developmental considerations are also key, as the trajectory-based subgroups suggest that evaluating individuals at a single time point may not be sufficient. Longitudinal lifespan studies are necessary to identify subgroups that could determine early predictors of later outcomes. Environmental factors, such as individual and sociodemographic variables should also be considered (Modabbernia et al., 2017). Ultimately, the goal of identifying sources of heterogeneity is to increase understanding of the underlying causes of autism and to improve the quality of life for autistic individuals and their families.

## Author Contributions

CWN, DSA, JL, MS, SR, and DGA contributed to the conception of the review. CWN wrote the first draft of the manuscript. DSA, PD, and PA wrote sections of the manuscript. BH and DSA conceptualized and designed the illustrations. All authors contributed to manuscript revision, read, and approved the submitted version.

## Conflict of Interest

DGA was on the Scientific Advisory Boards of Stemina Biomarkers Discovery, Inc. and Axial Therapeutics. The remaining authors declare that the research was conducted in the absence of any commercial or financial relationships that could be construed as a potential conflict of interest.

## Publisher’s Note

All claims expressed in this article are solely those of the authors and do not necessarily represent those of their affiliated organizations, or those of the publisher, the editors and the reviewers. Any product that may be evaluated in this article, or claim that may be made by its manufacturer, is not guaranteed or endorsed by the publisher.

## References

[B1] Agelink van Rentergem J. A. Deserno M. K. Geurts H. M. (2021). Validation strategies for subtypes in psychiatry: a systematic review of research on autism spectrum disorder. *Clin. Psychol. Rev.* 87:102033. 10.1016/j.cpr.2021.102033 33962352

[B2] Albano A. M. Silverman W. K. (1996). *The Anxiety Disorders Interview Schedule for Children for DSM-IV: Clinical Manual (Child and Parent Versions).* San Antonio, TX: Psychological Corporation.

[B3] Amaral D. G. Li D. Libero L. Solomon M. Van de Water J. Mastergeorge A. (2017). In pursuit of neurophenotypes: the consequences of having autism and a big brain. *Autism Res.* 10 711–722. 10.1002/aur.1755 28239961 PMC5520638

[B4] American Psychiatric Association (2013). *Diagnostic and Statistical Manual of Mental Disorders*, 5th Edn. Richmond, VA: American Psychiatric Association.

[B5] Andrews D. S. Lee J. K. Harvey D. J. Waizbard-Bartov E. Solomon M. Rogers S. J. (2021). A longitudinal study of white matter development in relation to changes in autism severity across early childhood. *Biol. Psychiatry* 89 424–432. 10.1016/j.biopsych.2020.10.013 33349451 PMC7867569

[B6] Andrews D. S. Lee J. K. Solomon M. Rogers S. J. Amaral D. G. Nordahl C. W. (2019). A diffusion-weighted imaging tract-based spatial statistics study of autism spectrum disorder in preschool-aged children. *J. Neurodev. Disord.* 11:32. 10.1186/s11689-019-9291-z 31839001 PMC6913008

[B7] Blume J. Wittke K. Naigles L. Mastergeorge A. M. (2021). Language growth in young children with autism: interactions between language production and social communication. *J. Autism Dev. Disord.* 51 644–665. 10.1007/s10803-020-04576-3 32588273

[B8] Breece E. Paciotti B. Nordahl C. W. Ozonoff S. Van de Water J. A. Rogers S. J. (2013). Myeloid dendritic cells frequencies are increased in children with autism spectrum disorder and associated with amygdala volume and repetitive behaviors. *Brain Behav. Immun.* 31 69–75. 10.1016/j.bbi.2012.10.006 23063420 PMC4229011

[B9] Buie T. Campbell D. B. Fuchs G. J. III Furuta G. T. Levy J. Vandewater J. (2010). Evaluation, diagnosis, and treatment of gastrointestinal disorders in individuals with ASDs: a consensus report. *Pediatrics* 125(Suppl. 1) S1–S18. 10.1542/peds.2009-1878C 20048083

[B10] Campbell D. J. Chang J. Chawarska K. (2014). Early generalized overgrowth in autism spectrum disorder: prevalence rates, gender effects, and clinical outcomes. *J. Am. Acad. Child Adolesc. Psychiatry* 53 1063.e–1073.e. 10.1016/j.jaac.2014.07.008 25245350 PMC4173120

[B12] Careaga M. Murai T. Bauman M. D. (2017a). Maternal immune activation and autism spectrum disorder: from rodents to nonhuman and human primates. *Biol. Psychiatry* 81 391–401. 10.1016/j.biopsych.2016.10.020 28137374 PMC5513502

[B11] Careaga M. Rogers S. Hansen R. L. Amaral D. G. Van de Water J. Ashwood P. (2017b). Immune endophenotypes in children with autism spectrum disorder. *Biol. Psychiatry* 81 434–441. 10.1016/j.biopsych.2015.08.036 26493496 PMC4788581

[B13] Courchesne E. Karns C. M. Davis H. R. Ziccardi R. Carper R. A. Tigue Z. D. (2001). Unusual brain growth patterns in early life in patients with autistic disorder: an MRI study. *Neurology* 57 245–254. 10.1212/wnl.57.2.245 11468308

[B14] De Meo-Monteil R. Nordahl C. W. Amaral D. G. Rogers S. J. Harootonian S. K. Martin J. (2019). Differential altered auditory event-related potential responses in young boys on the autism spectrum with and without disproportionate megalencephaly. *Autism Res.* 12 1236–1250. 10.1002/aur.2137 31157516 PMC7282708

[B15] Dellapiazza F. Vernhet C. Blanc N. Miot S. Schmidt R. Baghdadli A. (2018). Links between sensory processing, adaptive behaviours, and attention in children with autism spectrum disorder: a systematic review. *Psychiatry Res.* 270 78–88. 10.1016/j.psychres.2018.09.023 30245380

[B17] Dwyer P. De Meo-Monteil R. Saron C. D. Rivera S. M. (2021a). Effects of age on loudness-dependent auditory ERPs in young autistic and typically-developing children. *Neuropsychologia* 156:107837. 10.1016/j.neuropsychologia.2021.107837 33781752 PMC8102409

[B19] Dwyer P. Ferrer E. Saron C. D. Rivera S. M. (2021b). Exploring sensory subgroups in typical development and autism spectrum development using factor mixture modelling. Available online at: 10.1007/s10803-021-05256-6 (accessed September 27, 2021). 34499275 PMC9349169

[B16] Dwyer P. Saron C. D. Rivera S. M. (2020). Identification of longitudinal sensory subtypes in typical development and autism spectrum development using growth mixture modelling. *Res. Autism Spectr. Disord.* 78:101645. 10.1016/j.rasd.2020.101645 32944065 PMC7491753

[B18] Dwyer P. Wang X. De Meo-Monteil R. Hsieh F. Saron C. D. Rivera S. M. (2021c). Using clustering to examine inter-individual variability in topography of auditory event-related potentials in autism and typical development. *Brain Topogr.* 34 681–697. 10.1007/s10548-021-00863-z 34292447 PMC8436953

[B20] Gotham K. Pickles A. Lord C. (2012). Trajectories of autism severity in children using standardized ADOS scores. *Pediatrics* 130 e1278–e1284. 10.1542/peds.2011-3668 23090336 PMC3483889

[B21] Hazlett H. C. Poe M. Gerig G. Smith R. G. Provenzale J. Ross A. (2005). Magnetic resonance imaging and head circumference study of brain size in autism: birth through age 2 years. *Arch. Gen. Psychiatry* 62 1366–1376. 10.1001/archpsyc.62.12.1366 16330725

[B22] Hughes H. K. Mills Ko E. Rose D. Ashwood P. (2018). Immune dysfunction and autoimmunity as pathological mechanisms in autism spectrum disorders. *Front. Cell Neurosci.* 12:405. 10.3389/fncel.2018.00405 30483058 PMC6242891

[B23] Kerns C. M. Renno P. Kendall P. C. Wood J. J. Storch E. A. (2017). Anxiety disorders interview schedule–autism addendum: reliability and validity in children with autism spectrum disorder. *J. Clin. Child Adolesc. Psychol.* 46 88–100. 10.1080/15374416.2016.1233501 27925775 PMC5441235

[B24] Kerns C. M. Winder-Patel B. Iosif A. M. Nordahl C. W. Heath B. Solomon M. (2020). Clinically significant anxiety in children with autism spectrum disorder and varied intellectual functioning. *J. Clin. Child Adolesc. Psychol.* 50 780–795. 10.1080/15374416.2019.1703712 31971849 PMC9372909

[B25] Klein S. Sharifi-Hannauer P. Martinez-Agosto J. A. (2013). Macrocephaly as a clinical indicator of genetic subtypes in autism. *Autism Res.* 6 51–56. 10.1002/aur.1266 23361946 PMC3581311

[B26] Lai M.-C. Lombardo M. V. Auyeung B. Chakrabarti B. Baron-Cohen S. (2015). Sex/gender differences and autism: setting the scene for future research. *J. Am. Acad. Child Adolesc. Psychiatry* 54 11–24. 10.1016/j.jaac.2014.10.003 25524786 PMC4284309

[B27] Lee J. K. Amaral D. G. Solomon M. Rogers S. J. Ozonoff S. Nordahl C. W. (2020). Sex differences in the amygdala resting-state connectome of children with autism spectrum disorder. *Biol. Psychiatry Cogn. Neurosci. Neuroimaging* 5 320–329. 10.1016/j.bpsc.2019.08.004 31563470 PMC7033019

[B28] Lee J. K. Andrews D. S. Ozonoff S. Solomon M. Rogers S. Amaral D. G. (2021). Longitudinal evaluation of cerebral growth across childhood in boys and girls with autism spectrum disorder. *Biol. Psychiatry* 90 286–294. 10.1016/j.biopsych.2020.10.014 33388135 PMC8089123

[B29] Lehtinen M. K. Bjornsson C. S. Dymecki S. M. Gilbertson R. J. Holtzman D. M. Monuki E. S. (2013). The choroid plexus and cerebrospinal fluid: emerging roles in development, disease, and therapy. *J. Neurosci.* 33 17553–17559. 10.1523/JNEUROSCI.3258-13.2013 24198345 PMC3818536

[B30] Libero L. E. Nordahl C. W. Li D. D. Ferrer E. Rogers S. J. Amaral D. G. (2016). Persistence of megalencephaly in a subgroup of young boys with autism spectrum disorder. *Autism Res.* 9 1169–1182. 10.1002/aur.1643 27273931 PMC5292980

[B31] Libero L. E. Schaer M. Li D. D. Amaral D. G. Nordahl C. W. (2019). A longitudinal study of local gyrification index in young boys with autism spectrum disorder. *Cereb. Cortex* 29 2575–2587. 10.1093/cercor/bhy126 29850803 PMC6519847

[B32] Lin L.-Y. Huang P.-C. (2019). Quality of life and its related factors for adults with autism spectrum disorder. *Disabil. Rehabil.* 41 896–903. 10.1080/09638288.2017.1414887 29228834

[B33] Lord C. Risi S. Lambrecht L. Cook E. H. Leventhal B. L. DiLavore P. C. (2000). The autism diagnostic observation schedule-generic: a standard measure of social and communication deficits associated with the spectrum of autism. *J. Autism Dev. Disord.* 30 205–223. 11055457

[B34] Lord C. Rutter M. DiLavore P. C. Risi S. Gotham K. Bishop S. (2012). *Autism Diagnostic Observation Schedule*, 2nd Edn. Torrance, CA: Western Psychological Services.

[B35] Lord C. Rutter M. Le Couteur A. (1994). Autism diagnostic interview-revised: a revised version of a diagnostic interview for caregivers of individuals with possible pervasive developmental disorders. *J. Autism Dev. Disord.* 24 659–685. 10.1007/BF02172145 7814313

[B36] Maenner M. J. (2020). Prevalence of autism spectrum disorder among children aged 8 years — autism and developmental disabilities monitoring network, 11 sites, United States, 2016. *MMWR Surveill. Summ.* 69 1–12. 10.15585/mmwr.ss6904a1 32214087 PMC7119644

[B37] McConachie H. Wilson C. Mason D. Garland D. Parr J. R. Rattazzi A. (2020). What is important in measuring quality of life? Reflections by autistic adults in four countries. *Autism Adulth.* 2 4–12. 10.1089/aut.2019.0008 PMC8992842 36600984

[B38] McIntosh D. N. Miller L. J. Shyu V. Dunn W. (1999). Development and validation of the short sensory profile. *Sensory profile manual* 61 59–73.

[B39] Modabbernia A. Velthorst E. Reichenberg A. (2017). Environmental risk factors for autism: an evidence-based review of systematic reviews and meta-analyses. *Mol. Autism* 8:13. 10.1186/s13229-017-0121-4 28331572 PMC5356236

[B40] Mullen E. M. (1995). *Mullen Scales of Early Learning.* Circle Pines, MN: American Guidance Service, Inc.

[B41] Naigles L. R. Johnson R. Mastergeorge A. Ozonoff S. Rogers S. J. Amaral D. G. (2017). Neural correlates of language variability in preschool-aged boys with autism spectrum disorder. *Autism Res.* 10 1107–1119. 10.1002/aur.1756 28301102 PMC5548458

[B42] Nordahl C. W. Braunschweig D. Iosif A. M. Lee A. Rogers S. Ashwood P. (2013). Maternal autoantibodies are associated with abnormal brain enlargement in a subgroup of children with autism spectrum disorder. *Brain Behav. Immun.* 30 61–65. 10.1016/j.bbi.2013.01.084 23395715 PMC3641177

[B43] Nordahl C. W. Iosif A.-M. Young G. S. Hechtman A. Heath B. Lee J. K. (2020). High psychopathology subgroup in young children with autism: associations with biological sex and amygdala volume. *J. Am. Acad. Child Adolesc. Psychiatry* 59 1353.e–1363.e. 10.1016/j.jaac.2019.11.022 31972262 PMC7369216

[B44] Nordahl C. W. Iosif A.-M. Young G. S. Perry L. M. Dougherty R. Lee A. (2015). Sex differences in the corpus callosum in preschool-aged children with autism spectrum disorder. *Mol. Autism* 6:26. 25973163 10.1186/s13229-015-0005-4 PMC4429319

[B45] Nordahl C. W. Lange N. Li D. D. Barnett L. A. Lee A. Buonocore M. H. (2011). Brain enlargement is associated with regression in preschool-age boys with autism spectrum disorders. *Proc. Natl. Acad. Sci. U.S.A.* 108 20195–20200. 10.1073/pnas.1107560108 22123952 PMC3250128

[B46] Nordahl C. W. Mello M. Shen A. M. Shen M. D. Vismara L. A. Li D. (2016). Methods for acquiring MRI data in children with autism spectrum disorder and intellectual impairment without the use of sedation. *J. Neurodev. Disord.* 8 20–20. 10.1186/s11689-016-9154-9 27158271 PMC4858915

[B47] Nordahl C. W. Simon T. J. Zierhut C. Solomon M. Rogers S. J. Amaral D. G. (2008). Brief report: methods for acquiring structural MRI data in very young children with autism without the use of sedation. *J. Autism Dev. Disord.* 38 1581–1590. 10.1007/s10803-007-0514-x 18157624 PMC4864596

[B48] Ohta H. Nordahl C. W. Iosif A.-M. Lee A. Rogers S. Amaral D. G. (2016). Increased surface area, but not cortical thickness, in a subset of young boys with autism spectrum disorder. *Autism Res.* 9 232–248. 10.1002/aur.1520 26184828 PMC4886547

[B49] Onore C. Yang H. Van de Water J. Ashwood P. (2017). Dynamic Akt/mTOR signaling in children with autism spectrum disorder. *Front. Pediatr.* 5:43. 10.3389/fped.2017.00043 28361047 PMC5350147

[B50] Onore C. E. Nordahl C. W. Young G. S. Van de Water J. A. Rogers S. J. Ashwood P. (2012). Levels of soluble platelet endothelial cell adhesion molecule-1 and P-selectin are decreased in children with autism spectrum disorder. *Biol. Psychiatry* 72 1020–1025. 10.1016/j.biopsych.2012.05.004 22717029 PMC3496806

[B51] Patel S. Dale R. C. Rose D. Heath B. Nordahl C. W. Rogers S. (2020). Maternal immune conditions are increased in males with autism spectrum disorders and are associated with behavioural and emotional but not cognitive co-morbidity. *Transl. Psychiatry* 10:286. 10.1038/s41398-020-00976-2 32796821 PMC7429839

[B52] Restrepo B. Angkustsiri K. Taylor S. L. Rogers S. J. Cabral J. Heath B. (2020). Developmental-behavioral profiles in children with autism spectrum disorder and co-occurring gastrointestinal symptoms. *Autism Res.* 13 1778–1789. 10.1002/aur.2354 32767543 PMC7689713

[B53] Roberts T. P. L. Matsuzaki J. Blaskey L. Bloy L. Edgar J. C. Kim M. (2019). Delayed M50/M100 evoked response component latency in minimally verbal/nonverbal children who have autism spectrum disorder. *Mol. Autism* 10:34. 10.1186/s13229-019-0283-3 31428297 PMC6694560

[B54] Russell G. Mandy W. Elliott D. White R. Pittwood T. Ford T. (2019). Selection bias on intellectual ability in autism research: a cross-sectional review and meta-analysis. *Mol. Autism* 10:9. 10.1186/s13229-019-0260-x 30867896 PMC6397505

[B55] Rutter M. Bailey A. Lord C. (2003). *Social Communication Questionnaire (SCQ).* Los Angeles, CA: Western Psychological Services.

[B56] Salmond C. H. Vargha-Khadem F. Gadian D. G. de Haan M. Baldeweg T. (2007). Heterogeneity in the patterns of neural abnormality in autistic spectrum disorders: evidence from ERP and MRI. *Cortex* 43 686–699. 10.1016/s0010-9452(08)70498-2 17710821

[B57] Shen M. D. (2018). Cerebrospinal fluid and the early brain development of autism. *J. Neurodev. Disord.* 10 39–39. 10.1186/s11689-018-9256-7 30541429 PMC6292033

[B58] Shen M. D. Kim S. H. McKinstry R. C. Gu H. Hazlett H. C. Nordahl C. W. (2017). Increased extra-axial cerebrospinal fluid in high-risk infants who later develop autism. *Biol. Psychiatry* 82 186–193. 10.1016/j.biopsych.2017.02.1095 28392081 PMC5531051

[B59] Shen M. D. Nordahl C. W. Li D. D. Lee A. Angkustsiri K. Emerson R. W. (2018). Extra-axial cerebrospinal fluid in high-risk and normal-risk children with autism aged 2-4 years: a case-control study. *Lancet Psychiatry* 5 895–904. 10.1016/S2215-0366(18)30294-3 30270033 PMC6223655

[B60] Shen M. D. Nordahl C. W. Young G. S. Wootton-Gorges S. L. Lee A. Liston S. E. (2013). Early brain enlargement and elevated extra-axial fluid in infants who develop autism spectrum disorder. *Brain* 136 2825–2835. 10.1093/brain/awt166 23838695 PMC3754460

[B61] Soke G. N. Maenner M. J. Christensen D. Kurzius-Spencer M. Schieve L. A. (2018). Prevalence of co-occurring medical and behavioral conditions/symptoms among 4- and 8-year-old children with autism spectrum disorder in selected areas of the United States in 2010. *J. Autism Dev. Disord.* 48 2663–2676. 10.1007/s10803-018-3521-1 29524016 PMC6041136

[B62] Solomon M. Iosif A. M. Reinhardt V. P. Libero L. E. Nordahl C. W. Ozonoff S. (2018). What will my child’s future hold? Phenotypes of intellectual development in 2–8-year-olds with autism spectrum disorder. *Autism Res.* 11 121–132. 10.1002/aur.1884 29076255 PMC5961488

[B63] Sparks B. F. Friedman S. D. Shaw D. W. Aylward E. H. Echelard D. Artru A. A. (2002). Brain structural abnormalities in young children with autism spectrum disorder. *Neurology* 59 184–192. 10.1212/wnl.59.2.184 12136055

[B64] Waizbard-Bartov E. Ferrer E. Young G. S. Heath B. Rogers S. Wu Nordahl C. (2021). Trajectories of autism symptom severity change during early childhood. *J. Autism Dev. Disord.* 51 227–242. 10.1007/s10803-020-04526-z 32410098 PMC7810617

[B65] Williams Z. J. Abdelmessih P. G. Key A. P. Woynaroski T. G. (2021). Cortical auditory processing of simple stimuli is altered in autism: a meta-analysis of auditory evoked responses. *Biol. Psychiatry Cogn. Neurosci. Neuroimaging* 6 767–781. 10.1016/j.bpsc.2020.09.011 33229245 PMC8639293

[B66] Williams Z. J. Failla M. D. Gotham K. O. Woynaroski T. G. Cascio C. (2018). Psychometric evaluation of the short sensory profile in youth with autism spectrum disorder. *J. Autism Dev. Disord.* 48 4231–4249. 10.1007/s10803-018-3678-7 30019274 PMC6219913

[B67] Wittke K. Mastergeorge A. M. Ozonoff S. Rogers S. J. Naigles L. R. (2017). Grammatical language impairment in autism spectrum disorder: exploring language phenotypes beyond standardized testing. *Front. Psychol.* 8:532. 10.3389/fpsyg.2017.00532 28458643 PMC5394165

